# Background Subtraction Based on Color and Depth Using Active Sensors

**DOI:** 10.3390/s130708895

**Published:** 2013-07-12

**Authors:** Enrique J. Fernandez-Sanchez, Javier Diaz, Eduardo Ros

**Affiliations:** Department of Computer Architecture and Technology, ETSIIT, CITIC, University of Granada, C/ Periodista Rafael Gmez n02 2, E18071, Granada, Spain; E-Mails: jda@ugr.es (J.D.); eros@ugr.es (E.R.)

**Keywords:** background subtraction, foreground, segmentation, depth, Kinect, active sensors, computer vision, video surveillance, sensor fusion

## Abstract

Depth information has been used in computer vision for a wide variety of tasks. Since active range sensors are currently available at low cost, high-quality depth maps can be used as relevant input for many applications. Background subtraction and video segmentation algorithms can be improved by fusing depth and color inputs, which are complementary and allow one to solve many classic color segmentation issues. In this paper, we describe one fusion method to combine color and depth based on an advanced color-based algorithm. This technique has been evaluated by means of a complete dataset recorded with Microsoft Kinect, which enables comparison with the original method. The proposed method outperforms the others in almost every test, showing more robustness to illumination changes, shadows, reflections and *camouflage*.

## 1. Introduction

In recent years, there has been an increase of interest in the application of computer vision to video surveillance tasks. One of these tasks, which is typically considered the first step in video analytics systems, is the extraction of moving objects from a video sequence. A common approach for segmenting objects from the background is called *background subtraction*. This technique consists of analyzing a video sequence to create a reference background model and detect regions that belong to foreground objects.

Background subtraction is a well-known technique, which has aroused much interest as a research field. Therefore, there are many works in the literature focused on it: simple models for static backgrounds [1–3] or more advanced methods capable of dealing with dynamic backgrounds, such as MOG (Mixture of Gaussians) [4–6], Bayesian decision rules [7], the Codebook-based model [8,9], Kernel density estimation [10] or Component Analysis (PCA, Principal Component Analysis, and ICA, Independent Component Analysis) [11,12].

Despite current state-of-the-art algorithms being able to cope with classic issues (such as sudden and gradual illumination changes, moving background objects, repetitive movements, *etc.*), robustness is a critical requirement for video analytics. For that reason, many authors have proposed the fusion of different kinds of features, including intensity, edges and texture information [5,13–17]. However, these features are captured by the same kind of camera sensor, being thus affected by the same problems. In addition, due to the complexity of these methods, they require powerful processors to run in real-time. This makes them not suitable for embedded systems as smart cameras and decentralized camera networks.

In order to reduce the impact of issues related to camera sensors, we focus on the combined use of depth and color. Depth is an interesting cue for segmentation that is less affected by the classic color segmentation issues, such as shadows or highlighted regions. Depth information can be obtained in real-time by different methods or technologies: stereo-camera setups with disparity estimation algorithms [18], Time-of-Flight (ToF) cameras [19], Asus Xtion PRO [20] or the Kinect peripheral from Microsoft [21]. In our approach, we make use of the Kinect sensor, which offers high-resolution depth information with lower cost than Time-of-Flight cameras. The combination of depth and visual (RGB, Red-Green-Blue) sensing allows for more robust and accurate object detection.

Depth information has been used in foreground/background segmentation techniques by many authors [22–28]. Cristani *et al.* [28] proposes a comprehensive review of background subtraction techniques, focusing on different sensor channels, including systems based on stereo cameras. Some of the other works are focused on stereo vision algorithms [22–24], whilst the most recent ones focus on Time-of-Flight cameras [25–27]. Ivanov *et al.* [22] proposed an approach that warps one image of the pair in the other one by using disparity. If corresponding pixels do not match, they belong to a foreground object or to an *occlusion shadow*. The method in [22] does not use background subtraction algorithms. Gordon *et al.* [23] include disparity in an approximation of a mixture of Gaussians to model the background. The approach in [23] shares the hypothesis that by combining stereo and color, the effect of classic segmentation issues can be reduced. However, it fails to provide numerical evaluation of the quality of the method. Furthermore, the chosen approximation is unimodal, being unable to perform correctly in the presence of non-static backgrounds. Kolmogorov *et al.* [24] fused stereo and color/contrast information to perform background substitution for teleconferencing. The color/contrast model is composed by two Gaussians, one for the background process and another for the foreground. For that reason, it is a unimodal approach that suffers from the same issues as the previous work.

Crabb *et al.* [25], Zhu *et al.* [26] and Schiller *et al.* [27] focus on the combination of color and depth information obtained by low-resolution ToF cameras. Due to this low resolution (160 × 120, 176 × 144 and 204 × 204, respectively), efforts must be made to reduce inaccuracies, specially at object boundaries. In [25,26], foreground probability and likelihood are computed based on depth, and then, a trimap is generated classifying pixels on: definitely foreground, definitely background or uncertain. However, these methods are aimed at performing background substitution and are not well-suited for video surveillance. For example, Crabb *et al.* [25] requires defining a distance plane in which objects are accepted as foreground. This plane will not allow one to model scenarios where a foreground object is behind parts of the background, such as the scenarios belonging to the proposed dataset.

Schiller *et al.* [27] propose a method based on [23], which takes into account the reliability of the depth information. Depth maps are obtained by means of a ToF-camera, at a resolution of 204 × 204 pixels. Our approach uses reliability measure provided by the sensor, and depth information is obtained at much higher resolution, thus directly reducing inaccuracies. Results show that the proposed background subtraction algorithm and fusion methods allow us to obtain higher quality foreground masks.

In this work, we propose an adaptation of the Codebook background subtraction algorithm [8], which fuses depth and color information to segment foreground regions, focused on video analytics. Although other authors have already studied the inclusion of depth cues in background subtraction models, the presented work is innovative in different points:

- It uses a high performance and low cost depth sensor, which directly provides accurate and dense depth estimations.
- It is based on the Codebook model, which has been naturally generalized to integrate depth estimations. This model offers a good trade-off between accuracy and efficiency and can be reasonably extended to use more kinds of features [9,29,30]. We propose a novel method to integrate depth and color information. This technique uses depth cues to bias the segmentation based on color.
- We provide an extensive qualitative and quantitative study, based on benchmark sequences that are made available to facilitate future comparisons. This study allows us to quantify the improvement obtained in different scenarios, which are complicated for color-based techniques, but also for depth estimation sensors.

The paper is organized as follows. In Section 2, we briefly describe the original Codebook model. In Section 3, the adaptation of the Codebook model to integrate depth and color information is explained. We present, in Section 4, the provided dataset, and results are shown and analyzed. Finally, conclusions and a discussion are presented in Section 5.

## 2. Codebook Background Subtraction Model

The Codebook algorithm, as proposed by Kim *et al.* [8], constructs a background model based on a quantization/clustering method described by Kohonen [31] and Ripley [32]. According to these works, the background model for each pixel is composed of a codebook consisting of one or more codewords. A codeword is a data structure that contains information not only about color and brightness, but also about frequency of access to its contents, allowing one to maintain a trace of use of the codeword.

Essentially, the Codebook algorithm consists of three different stages: construction of the initial codebook, foreground detection and model maintenance. All these stages are properly described in [8]. Therefore, in this work, we describe the basics of the mathematical model and the computation of codebooks, so that the extension to the usage of depth information can be more easily followed.

### 2.1. Model Construction

Given a set of *N* frames, a training sequence, *S*, is used for each pixel consisting of *N* RGB vectors: *S* = {*v*_1_,*v*_2_,*…v_N_*}. Initially, each pixel has an associated codebook, represented as *C* = {*c*_1_, *c*_2_,*c*_3_,*…c_L_*}, consisting of *L* codewords. The number of codewords for each pixel may be different. Each codeword, *c_i_*, *i* = 1*…L;*, consists of an RGB vector, *v_i_*= (*R̅_i_*, *G̅_i_*, *B̅_i_*), and a six-tuple $aux_{i}=\langle I_{\text{min}}^{i},I_{\text{max}}^{i},f_{i},\lambda_{i},p_{i},q_{i}\rangle$. The tuple, *aux_i_*, contains intensity values and temporal variables as described below:

- *v_i_*= (*R̅_i_*, *G̅_i_*, *B̅_i_*), average value of each color component.
- $I_{\text{min}}^{i}$, $I_{\text{max}}^{i}$, minimum and maximum brightness, respectively, of all pixels assigned to codeword, *c_i_*.
- *f_i_*, the frequency with which codeword *c_i_* has been.
- *λ_i_*, the maximum negative run-length (MNRL), defined as the longest interval of time during which codeword *c_i_* has not been updated.
- *p, q*, the first and last updating access times of codeword *c_i_*.

Some of the values of the codeword (*λ_i_*,*p*, *q*) are only used to deal with the presence of foreground objects during the construction. Since this mechanism is explained in detail in [8], we focus on the use of color and brightness variables.

A reduced pseudo-code for the codebook construction stage is given in Algorithm 1.


**Algorithm 1:** Algorithm for codebook construction
 *C* ← *ϕ* **for**
*t*= 1 → *N*
**do**  *x_t_* = (*R*, *G*, *B*), 
$I\leftarrow \sqrt{R^{2}+G^{2}+B^{2}}$  Find the codeword, *c_m_*, in *C* matching to *x_t_* based on two conditions:  (a) *colordist*(*x_t_*, *v_m_*) ≤ *ϵ*_1_  (b) *brightness* (*I*, 〈
$I_{\text{min}}^{m}$, 
$I_{\text{max}}^{m}$〉) = **true**  **if**
*C* = *ϕ*
**or** there is no match **then**   {Create new codeword and add it to *C*}  **else**   {Update matched codeword}  **end if** **end for**


According to the description of the algorithm, the two conditions, (a) and (b), detailed in Equations (2) and (4), are satisfied when the values of pixel *x_t_* and *c_m_* are similar, both in chromaticity and brightness intensity. In order to deal with global and local illumination changes, color distortion and brightness distortion are evaluated independently. When there is an input pixel, *x_t_*, and a codeword, *c_i_*, with *v_i_*= (*R̅_i_*, *G̅_i_*, *B̅_i_*):

(1) $$\begin{array}{ll} {\left\|x_{t}\right\|}^{2} & =R^{2}+G^{2}+B^{2}\\ {\left\|v_{i}\right\|}^{2} & ={{\bar{R}}_{i}}^{2}+{{\bar{G}}_{i}}^{2}+{{\bar{B}}_{i}}^{2}\\ {\langle x_{i},v_{i}\rangle }^{2} & ={\left({\bar{R}}_{i}R+{\bar{G}}_{i}G+{\bar{B}}_{i}B\right)}^{2} \end{array}$$

The color distortion, *δ*, can be calculated by Equation (2):

(2) $$\begin{array}{lll} p^{2} & = & {\left\|x_{t}\right\|}^{2}\cos^{2}\theta =\frac{{\langle x_{t},v_{i}\rangle }^{2}}{{\left\|x_{t}\right\|}^{2}}\\ \text{colordist}\;(x_{t},v_{i}) & = & \delta =\sqrt{{\left\|x_{t}\right\|}^{2}-p^{2}} \end{array}$$

In order to allow for adaptation to brightness changes, *I_min_* and *I_max_* are stored in the codeword information. Brightness is allowed to vary in a certain range, [*I_low_, I_hi_*], defined as:

(3) $$\begin{array}{lll} I_{\text{low}} & = & \alpha I_{\text{max}}\\ I_{\text{hi}} & = & \text{min}\;\left\{\beta I_{\text{max}},\frac{I_{\text{min}}}{\alpha }\right\} \end{array}$$

Typically, *α* is in the interval, [0.4, 0.8], and *β* is in the interval, [1.1, 1.5]. The brightness function is defined in Equation (4).

(4) $$\text{brightness}\;(I,\langle I_{\text{min}},I_{\text{max}}\rangle )=\{\begin{array}{ll} \text{true} & ifI_{\text{low}}\leq \left\|x_{t}\right\|\leq I_{\text{hi}}\\ \text{false} & \text{otherwise} \end{array}$$

During the *foreground detection* stage, color and brightness distortions between each input pixel and the model are computed. Subsequently, the pixel is matched against a codeword based on the two conditions, and it is classified in the foreground or background according to Equation (5):

(5) $$\text{BGS}(x)=\{\begin{array}{ll} \text{BG} & \text{if}\;(\text{colordist}\;(x_{t},v_{i})<\epsilon )\wedge \\ & \text{brightness}\;(I,\langle I_{\text{min}},I_{\text{max}}\rangle )\\ \text{FG} & \text{otherwise} \end{array}$$

## 3. Depth-Extended Codebook: DECB

The fusion of background subtraction models with stereo models for disparity computation has been previously studied by Gordon *et al.* [23], improving the performance obtained by each separate technique. In [23] a four-channel background subtraction algorithm based on a unimodal mixture of Gaussians [4] is proposed.

In our contribution, we have studied the integration of depth information with RGB values based on the Codebook model [8]. Our approach consists of an update of the model proposed by Gordon *et al.*, although in our case, a four-channel (*R, G, B, Z*) codebook has been used. The inclusion of depth information in our model is performed in two different ways: the first one considers depth as the fourth channel of the codebook, which has an independent mechanism from color and brightness; the second one biases the distance in chromaticity associated to a pixel according to the depth distance.

Our approach to RGB-D (RGB and Depth) background subtraction is the generalization of the Codebook model proposed by Kim *et al.* [8], described in Section 2, to work with depth values as a fourth channel. The Depth-Extended Codebook works by enhancing the matching conditions between an input pixel value and a codeword. In the original algorithm, the pixel value matches the codeword if both color and brightness distortions are below a threshold Equations (2) and (4). Our approach includes additional conditions based on depth. Since depth information is one-dimensional, we have considered the evaluation of matching between the pixel value and the background model using a method similar to the brightness condition.

(6) $$\begin{array}{lll} D_{\text{low}} & = & \alpha_{D}D_{\text{max}}\\ D_{hi} & = & \text{min}\;\left\{\beta_{D}D_{\text{max}},\frac{D_{\text{min}}}{\alpha_{D}}\right\} \end{array}$$

In Equation (7), we obtain a range of values, [*D_low_*, *D_hi_*], which represents the depth change allowed for input values. *D_low_* and *D_hi_* are computed from *D_min_* and *D_max_*, which are the minimum and maximum depth values for a codeword. These two values are added to the six-tuple described in the original model (Section 2.1). *α_D_* and *β_D_* define the threshold in the depth distortion, being typically *α_D_* between 0.4 and 0.7 and *β_D_* between 1.1 and 1.5. The logical disparity function is defined as follows:

(7) $$\text{disparity}(D,\langle D_{\text{min}},D_{\text{max}}\rangle )=\{\begin{array}{ll} \text{true} & \text{if}\neg \text{Valid}(D)\vee \\ & (D_{\text{low}}\leq D\wedge \\ & D\leq D_{hi)}\\ \text{false} & \text{otherwise} \end{array}$$

When color, brightness and disparity distortions have been computed, the algorithm matches the current pixel value with the appropriate codeword based on these conditions.

Our approach aims to improve the robustness of the color-based algorithm to shadows, highlighted regions and sudden lighting changes. Depth computation sensors are more robust to lighting artifacts and shadows than passive sensors, such as cameras, since they work at the infrared range without interferences with visible light. For that reason, instead of simply considering depth as an independent fourth channel, deeper dependence between RGB and depth has been studied.

**Figure 1.**
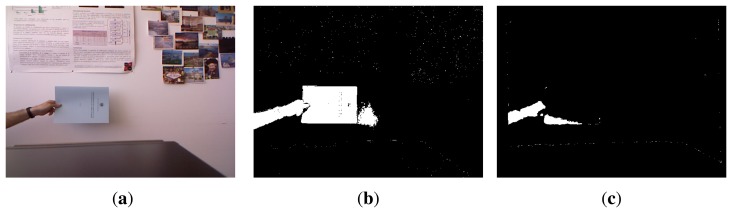
Example of complicated scenario for RGB-D methods: presence of shadows and flat foreground objects. Foreground objects are correctly detected by color-based algorithms, while they are misdetected by depth-based ones, since the objects are too close to the wall to be discernible. (**a**) Original frame; (**b**) RGB Detection; (**c**) Depth Detection.

The most straightforward method to remove shadows and highlighted regions will be not considering color distortion if the pixel is the background according to depth information. However, Figure 1 shows a scenario where this approach would produce misdetections, due to the presence of foreground objects with similar depth to the background.

Our approach consists of modifying the condition around color distortion to consider depth when color distortion is between two thresholds, *ϵ*_1_ and *ϵ*_2_. This second threshold, *ϵ*_2_, is fixed to 1.6*ϵ*_1_. Thus, a pixel is classified as foreground or background, as in Equation (8):

(8) $$\text{BGS}(x)=\{\begin{array}{ll} \text{BG} & \text{if}\;(\text{colordist}(x,c_{m}))\leq \epsilon_{1}\vee \\ & (\epsilon_{1}<\text{colordist}(x,c_{m})\leq \epsilon_{2}\wedge \;\text{disparity}(D,\langle D_{\text{min}},D_{\text{max}}\rangle )))\wedge \\ & \text{brightness}\;(I,\langle I_{\text{min}},I_{\text{max}}\rangle )\wedge \\ & \text{disparity}\;(D,\langle D_{\text{min}},D_{\text{max}}\rangle )\\ \text{FG} & \text{otherwise} \end{array}$$

According to Equation (7), the condition, *disparity* (*D*, 〈*D_min_*, *D_max_*〉), is true if the depth value of the pixel obtained by the active sensor is *invalid*. Therefore, when the depth value is invalid, the condition required in Equation (8) depends entirely on *colordist* (*x_t_*, *v_i_*) and *brightness* (*I*, 〈*I_min_*, *I_max_*〉), relying on the color-based background model for the foreground/background classification.

Equation (8) can be interpreted in the following way: if an input pixel is considered to be foreground, but it is close enough to the threshold, the classification will take into account the knowledge about the depth value for that pixel.

This modification will produce less foreground pixels than the 4D codebook without biasing the color threshold, most of the removed pixels being false positives in the original model. Section 4 shows the experiments performed and the results obtained with both RGB-D algorithms, as well as the color-based codebook.

## 4. Experiments and Results

This section describes the experiments performed to test the proposed methods and compare them with the original Codebook algorithm. We explain the dataset and metrics used to evaluate different approaches and the parameter settings for our method. Furthermore, a quantitative and qualitative analysis of the results is performed.

### 4.1. Dataset and Metrics

In order to evaluate objectively these algorithms by means of a quantitative analysis, we require the use of a dataset with ground truth segmentation. There are different benchmarks used for evaluation of background models [33,34], but they do not have available information about depth. On the other hand, there are benchmarks focused on the use of depth to recognize human activities [35]. However, this kind of benchmark does not provide ground truth for background subtraction models, but a set of different categories of activities to classify into. Thus, since we are focused on the use of consumer depth sensors, we have recorded and manually segmented some sequences by using Kinect [21], although any kind of active sensor would have been appropriate, too (ASUS Xtion PRO [20] or ToF-cameras [19]). Data from Kinect have been obtained by using OpenCV [36] and OpenNI drivers [37]. The recorded sequences have been publicly available at [38]. The sequences are the following:

- *ChairBox*: a person enters the field of view and leaves a box on a chair. There are flickering lights, as well as areas where depth cannot be obtained by infrared active sensors.
- *Wall*: a flat object (paper sheet) appears close to a wall, creating shadows and highlighted regions. The main difficulties are the similarity of depth between foreground and background and the change of lighting.
- *Shelves*: a person enters the scene and puts two objects on shelves. There are changes of exposure, as well as difficult depth estimation.
- *Hallway*: sequence recorded aiming at a hallway. There are reflections, complicated lighting, objects similar to the background and sudden illumination changes.

In order to evaluate background subtraction models, relative measures have been calculated based on true and false positives and negatives (*TP*, *FP*, *TN*, *FN*). These measures are widely used in the literature [2,39] and are defined as follows: *recall* is the true positive rate, *R = TP*/(*TP* + *FN*); *precision* is the ratio between the number of correctly detected pixels and the total number of pixels marked as foreground, *P = TP/*(*TP* + *FP*); finally, one accuracy metric, *F*_1_, which combines *precision* and *recall* to evaluate the quality of the segmentation. The *F*_1_ measure is defined as follows:

(9) $$F_{1}=2\cdot \frac{P\cdot R}{P+R}$$

This measure offers a trade-off between the ability of an algorithm to detect foreground and background pixels. This allows for the general evaluation of the robustness of the algorithm. In general, the higher the value of this estimator, the better the performance, although it usually requires an additional qualitative analysis to explain the numerical results.

### 4.2. Parameter Settings

The proposed approach consists of several parameters that define its behavior. Since we want to evaluate the overall performance of the algorithms, we have chosen a unique set of parameters that gives good enough results on the complete dataset. Table 1 shows the values of these parameters:

### 4.3. Performance Evaluation

By using the previously mentioned sequences, six different approaches have been studied and evaluated. These approaches are the following ones: a 4D version of MOG based on the implementation proposed by Schiller *et al.* [27] (MOG4D), the Pixel-Based Adaptive Segmenter [40] (PBAS), the original color-based Codebook (CB), the Codebook based only on depth (CB1D), the 4D Codebook (CB4D) without bias over color threshold and the Depth-Extended Codebook (DECB). The tested version of MOG4D differs slightly from that proposed by Schiller *et al.* [27], since we cannot use the amplitude image provided by the ToF-camera. For that reason, the fusion of color and range has been performed according to Gordon *et al.* [23], as a disjunction of the previous results.

The experiments performed on Codebook-based approaches involve only the segmentation stage, without morphological filtering. We have decided to avoid any post-processing stage to evaluate the capabilities of the algorithms by themselves, although raw results can be easily improved by these simple operators. In addition, morphological filtering can be applied after segmentation in any moment. Nevertheless, the MOG4D approach includes morphological filtering, as in the approach proposed by Gordon *et al.* [23], in order to remove small isolated foreground points caused by noise.

**Figure 2.**
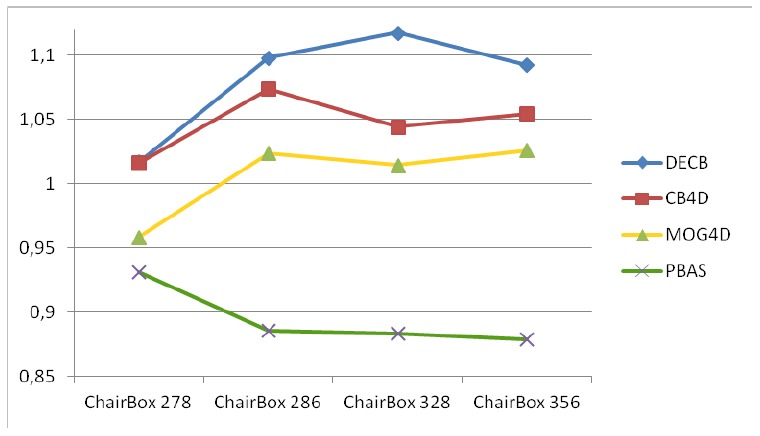
*F*_1_ gain over standard (color-based) CB obtained from the test, ChairBox.

Figure 2 and Table 2 show the quantitative results obtained in the ChairBox sequence. Table 2 shows *F*_1_ values resultant from the five approaches on the evaluation frames, the mean and standard deviation. Figure 2 shows the *gain* on *F*_1_ obtained by the three RGB-D algorithms (MOG4D, CB4D and DECB) and the Pixel-Based Adaptive Segmenter [40] (PBAS) over the color-based one (CB). All RGB-D approaches get improvements against CB, obtaining higher *F*_1_ values despite the good performance of the color-based method. This good performance explains why the *gain* is moderate, since the *gain* is limited by $1/F_{1}^{CB}$, where $F_{1}^{CB}$ is the *F*_1_ value obtained by the CB algorithm (for example, when $F_{1}^{CB}=0.845$, *gain* ≤ 1.183). PBAS obtains worse results than CB, because of misdetections in the darkest regions of the image. The graph shows that The Depth-Extended Codebook obtains the best results in all tests, whilst MOG4D gets more moderate results than the Codebook-based approaches.

**Figure 3.**
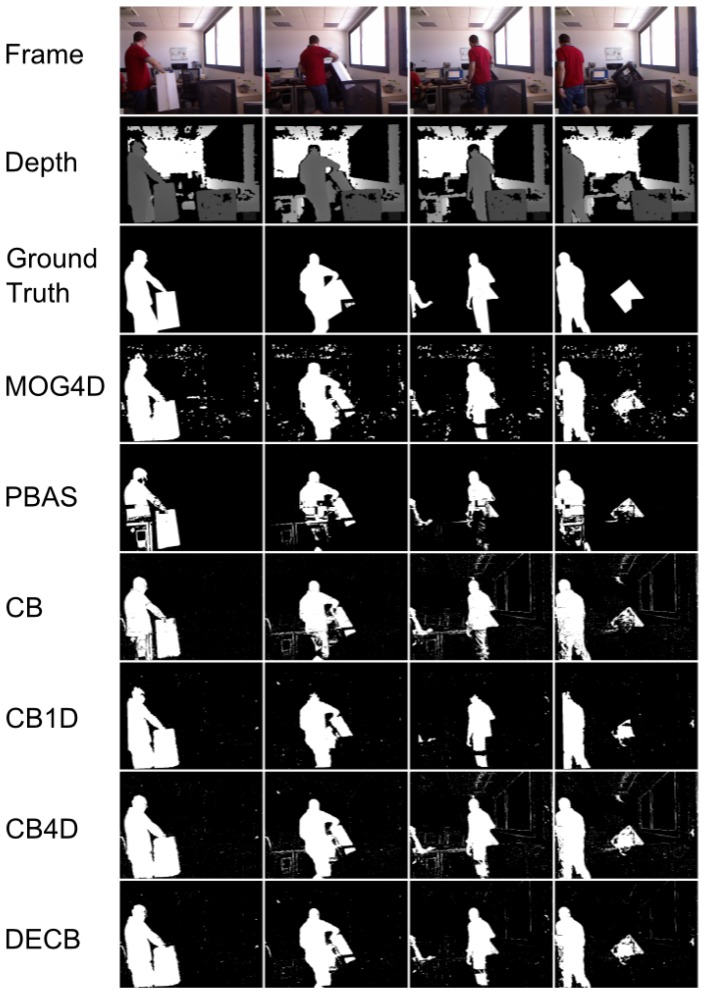
Results obtained from the test, ChairBox. 4D version of Mixture of Gaussians (MOG4D) includes a morphological opening stage, whilst Codebook-based approaches do not perform it, producing, then, more noise, due to isolated pixels. Most of this noise is filtered by the Depth-Extended Codebook (DECB) by means of the fusion method.

Figure 3 shows the segmentation produced by the five approaches. In general, the CB4D algorithm improves over CB and CB1D by using depth and color, but DECB reduces the amount of noise generated by both algorithms (specially noticeable on the last two frames).

**Figure 4.**
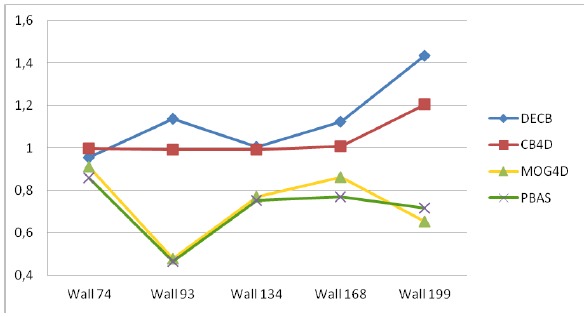
*F*_1_ gain over CB obtained from the test, Wall.

**Figure 5.**
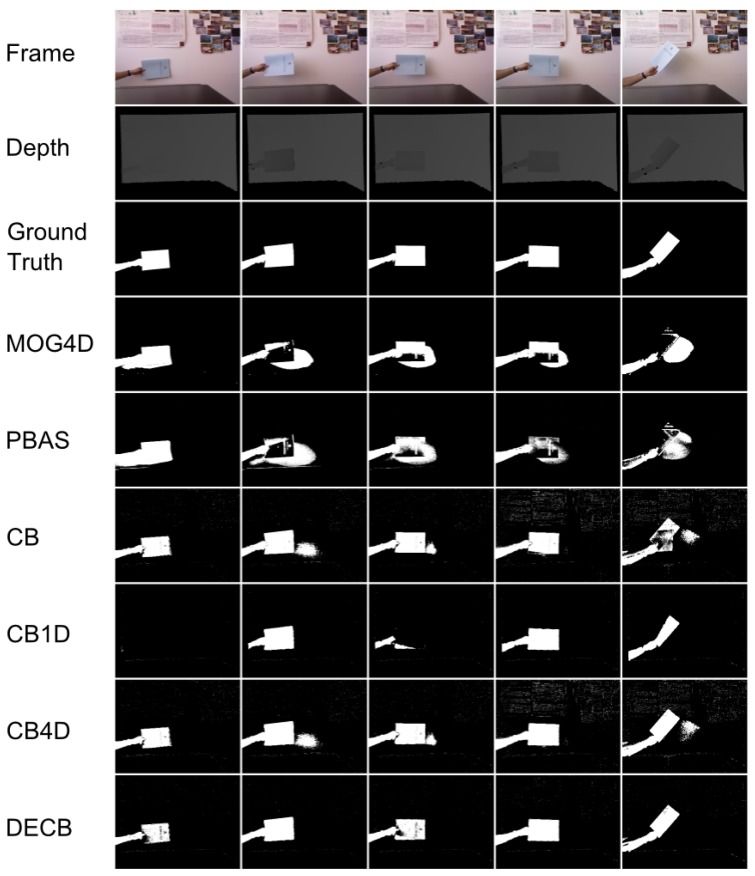
Results obtained from the test, Wall.

The second sequence, Wall, is especially complicated for the depth-based algorithm, due to similar depth between foreground objects and background. This is shown in Figure 4, where MOG4D obtains worse results than CB in all tests, whilst the Depth-Extended Codebook obtains slightly worse results than CB in one frame. This can be explained by checking Figure 5, where, in the first frame, the CB1D approach is unable to detect the object, thus misleading the 4D Codebook. However, despite being based on useless data, DECB gets *F*_1_ = 0.9 (Table 3), showing that it is fairly robust to difficult situations.

In addition, it gets much better results on every other frame, reaching *gain* values over 40%. The last two frames in Figure 5 show the reasons of this *gain*, which are proficient noise reduction and a complete shadow suppression by using depth values.

**Figure 6.**
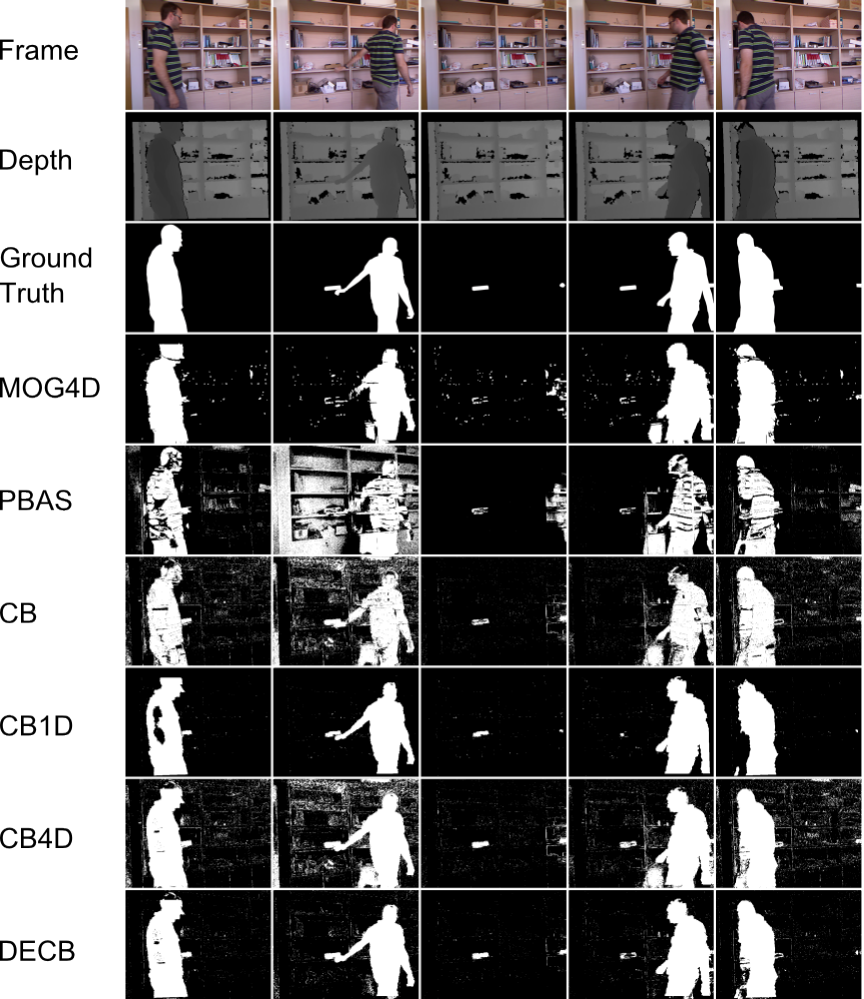
Results obtained from the test, Shelves.

In the third sequence, Shelves, the main difficulty is related to changes of lighting and exposure that produce many false positives on the entire image. This can be seen in Table 4 with the decrease of *F*_1_ obtained by the CB approach, as well as in Figure 6, with the big amount of noise on the furniture. PBAS does work on each channel separately, not dividing color and brightness, being thus prone to errors in the presence of global illumination changes and cast shadows. In addition, PBAS adapts more slowly to false positives, since it updates foreground pixels with less probability.

**Figure 7.**
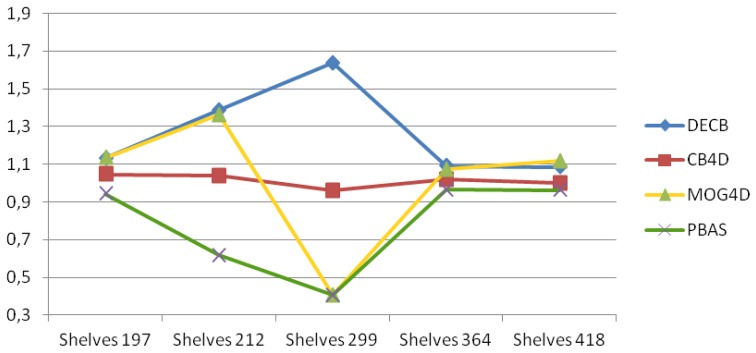
*F*_1_ gain over CB obtained from the test, Shelves.

Depth is a more stable cue, although there are regions too close to the sensor to be estimated, as well as foreground objects too close to the background. Figure 7 shows that the DECB algorithm obtains much better results by using depth and color combined, since each different input can overcome the weakness of the other. MOG4D gets very good results in four frames, although it is prone to errors, due to noise in frame 299. In this graph, *gain* values between 10% and more than 60% are obtained by DECB in all tests of the sequence, proving that the proposed method is much more robust than the original one based only on color cues.

**Figure 8.**
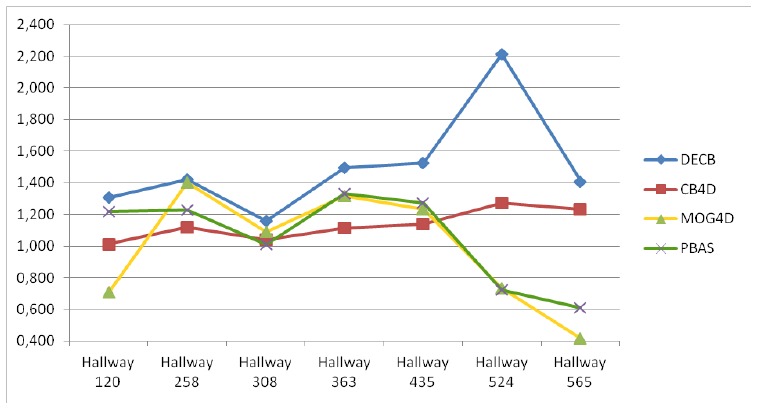
*F*_1_ gain over CB obtained from the test, Hallway.

Figure 8 and Table 5 show the results for the last sequence, Hallway. This sequence being especially complicated, due to the amount of difficulties, *F*_1_ values for the CB algorithm are quite low, which allows for higher possible *gain* values (higher improvement), as seen in Figure 8. According to this graph, both CB4D and DECB approaches offer improvement over the original algorithm, but the latter gets much greater *gain* values (up to 120% in one test). MOG4D and PBAS show good results in most of the frames, but perform worse than the others in the presence of sudden illumination changes.

**Figure 9.**
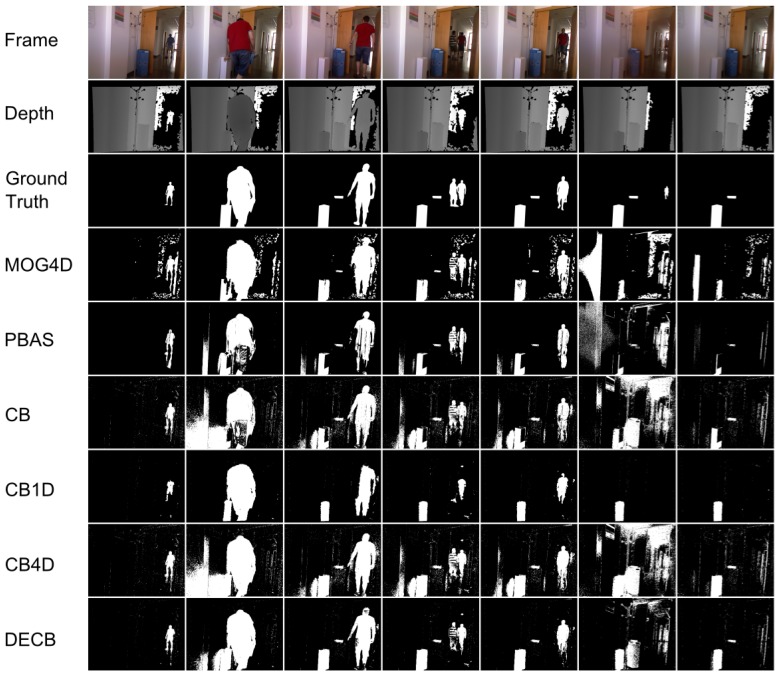
Results obtained from the test, Hallway.

By checking Figure 9, more detailed qualitative analysis can be performed. In general, it is shown that the DECB algorithm gets an important noise reduction, as well as almost total shadow suppression. In addition, the presence of objects with similar color to the background is complicated for the CB approach, but solved with the usage of depth information. This also happens on the fifth frame, with reflections on the floor that are detected correctly by the CB1D and DECB approaches. The most complicated frame in this sequence, that is, the sixth evaluation frame, includes sudden illumination changes. A directional light is turned on, producing changes in a big region of the image. Since the CB1D approach is based only on depth obtained by infrared sensors from the depth camera, it does not suffer from this lighting change. For that reason, despite the CB and CB4D approaches having a considerable amount of false positives, DECB minimizes this amount, thus being more robust than the other methods.

**Figure 10.**
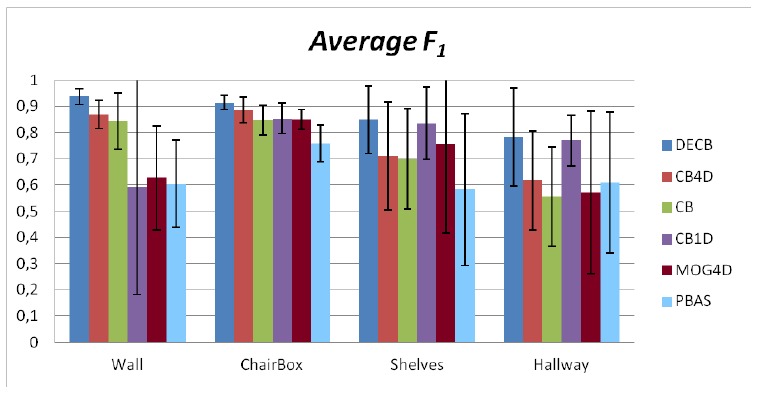
Average *F*_1_ obtained from the entire benchmark, including error bars showing the standard deviation (along each benchmark sequence).

Finally, Figure 10 shows the average *F*_1_ obtained by each approach in each sequence of the entire benchmark, while error bars show the standard deviation. According to this figure, the Depth-Extended Codebook (DECB) shows the best results on every sequence, and the standard deviation associated with this approach is lower than any other, which is a sign of its robustness. Only in one case, the CB1D algorithm has lower standard deviation, because of the change of illumination in the Hallway sequence, but even in this case, the Depth-Extended Codebook outperforms the other algorithm.

## 5. Conclusions

In this work, we have analyzed the fusion of depth and color to perform background subtraction. Depth information has been obtained by means of a consumer depth sensor, which allows for high-resolution depth maps at a lower cost than Time-of-Flight cameras. In addition, since depth is obtained by using infrared structured light instead of image processing, both signals are complementary and can be used to tackle classical issues of background subtraction algorithms.

We propose an adaptation of the Codebook algorithm [8] to use depth, as well as color. The Codebook algorithm is an advanced multimodal method that offers good trade-off between accuracy and efficiency, which makes it a very appropriate approach for implementation on embedded systems and smart cameras. Furthermore, it is robust to dynamic background and gradual scene changes. The use of depth enables proficient shadow suppression, as well as reduction of noise, due to sudden illumination changes. In addition, it minimizes the impact of *camouflage* (foreground objects with color similar to background).

We have studied two different approaches that differ in the depth integration method: the first one simply considers depth as a fourth channel of the background model, while the second one adds a joint RGB-D fusion method. Qualitative and quantitative analysis have been performed by using a complete dataset recorded with Kinect, which is made publicly available at [38]. Results show a considerable improvement on accuracy and robustness when using depth and color combined, since the proposed approach outperforms the other methods in almost every test. This is especially relevant taking into account that the chosen sequences are complicated and present typical cases where background subtraction methods fail. Therefore, our methods clearly increase the robustness of this segmentation stage.

Regarding computational costs, the selected model is efficient, since the costs associated with depth estimation are removed by the use of active sensors. In addition, the color-based algorithm has been previously implemented in real-time on FPGA (Field-Programmable Gate Array) [9], the Depth-Extended Codebook being suitable for embedded systems and smart cameras.

Future work will include use of depth in other video surveillance tasks, such as tracking, calibration and multi-camera setups.

## Figures and Tables

**Table 1.** Parameters selected for the proposed approaches.

**Parameter**	**Value**	**Parameter**	**Value**
*ϵ*_1_	10	*ϵ*_2_	1.6*ϵ*_1_
*α*	0.75	*β*	1.3
*α_D_*	0.75	*β_D_*	1.25
*tTrain*	50	*T_H_*	40
*T_add_*	100	*T_delete_*	100

**Table 2.** Segmentation evaluation for sequence, ChairBox. The table shows *F*_1_ results for the five studied approaches on four different evaluation frames, the mean and standard deviation on the entire sequence.

**ChairBox**	**Evaluation Frame**				**Global**	

**Approach**	**278**	**286**	**328**	**356**	*μ*	*σ*
DECB	**0.937**	**0.928**	**0.876**	**0.914**	**0.914**	0.027
CB4D	0.936	0.907	0.819	0.882	0.886	0.050
CB	0.921	0.845	0.784	0.837	0.847	0.057
CB1D	0.904	0.904	0.800	0.808	0.854	0.058
MOG4D	0.883	0.865	0.795	0.859	0.851	0.038
PBAS	0.858	0.749	0.693	0.736	0.759	0.070

**Table 3.** Segmentation evaluation for sequence, Wall. The table shows *F*_1_ results for the five studied approaches on five different evaluation frames, the mean and standard deviation on the entire sequence.

**Wall**	**Evaluation Frame**					**Global**	

**Approach**	**74**	**93**	**134**	**168**	**199**	***μ***	***σ***
DECB	0.900	**0.966**	**0.912**	**0.957**	**0.952**	**0.938**	0.029
CB4D	0.939	0.843	0.901	0.857	0.800	0.868	0.054
CB	**0.942**	0.850	0.910	0.851	0.664	0.843	0.108
CB1D	0.006	0.927	0.314	0.919	0.806	0.595	0.414
MOG4D	0.860	0.406	0.699	0.734	0.435	0.627	0.198
PBAS	0.808	0.395	0.684	0.654	0.476	0.604	0.166

**Table 4.** Segmentation evaluation for sequence, Shelves. The table shows *F*_1_ results for the five studied approaches on five different evaluation frames, the mean and standard deviation on the entire sequence.

**Shelves**	**Evaluation Frame**					**Global**	

**Approach**	**197**	**212**	**299**	**364**	**418**	***μ***	***σ***
DECB	0.926	0.909	**0.622**	**0.876**	0.909	**0.848**	0.128
CB4D	0.855	0.681	0.365	0.819	0.837	0.711	0.205
CB	0.818	0.655	0.380	0.804	0.838	0.699	0.192
CB1D	0.897	**0.942**	0.595	0.863	0.876	0.835	0.137
MOG4D	**0.927**	0.892	0.154	0.862	**0.937**	0.754	0.337
PBAS	0.771	0.405	0.155	0.775	0.807	0.583	0.291

**Table 5.** Segmentation evaluation for sequence, Hallway. The table shows *F*_1_ results for the five studied approaches on seven different evaluation frames, the mean and standard deviation on the entire sequence.

**Hallway**	**Evaluation Frame**							**Global**	

**Approach**	**120**	**258**	**308**	**363**	**435**	**524**	**565**	***μ***	***σ***
DECB	0.782	0.888	**0.930**	**0.844**	**0.905**	0.385	**0.745**	**0.783**	0.187
CB4D	0.606	0.701	0.835	0.629	0.675	0.222	0.653	0.617	0.190
CB	0.598	0.625	0.802	0.565	0.593	0.174	0.529	0.555	0.189
CB1D	**0.791**	**0.939**	0.791	0.630	0.801	**0.693**	0.744	0.770	0.097
MOG4D	0.424	0.875	0.875	0.744	0.732	0.128	0.221	0.571	0.311
PBAS	0.730	0.768	0.812	0.752	0.754	0.126	0.322	0.609	0.270
